# Patient and Hospital Characteristics Associated with Admission Among Patients With Minor Isolated Extremity Firearm Injuries: A Propensity-Matched Analysis

**DOI:** 10.1097/AS9.0000000000000430

**Published:** 2024-05-06

**Authors:** Arielle C. Thomas, Regina Royan, Avery B. Nathens, Brendan T. Campbell, Susheel Reddy, Sarabeth Spitzer, Doulia Hamad, Angie Jang, Anne M. Stey

**Affiliations:** From the * Department of Surgery, Medical College of Wisconsin, Milwaukee, WI; † American College of Surgeons, Chicago, IL; ‡ Department of Emergency Medicine, Feinberg School of Medicine, Northwestern University, Chicago, IL; § Department of Surgery, Sunnybrook Health Sciences Center and the University of Toronto, Toronto, ON, Canada; ‖ Department of Pediatric Surgery, Connecticut Children’s Medical Center and University of Connecticut School of Medicine, Hartford, CT; ¶ Department of Medicine, Feinberg School of Medicine, Northwestern University, Chicago, IL; # Department of Surgery, Center for Surgery and Public Health, Brigham and Women’s Hospital, Harvard Medical School, Boston, MA; ** Department of Surgery, Feinberg School of Medicine, Northwestern University, Chicago, IL.

**Keywords:** firearm injury, insurance, insurance and firearm injury, insurance and hospital admission, minor isolated extremity firearm injury, trauma

## Abstract

**Objective:**

To quantify the association between insurance and hospital admission following minor isolated extremity firearm injury.

**Background:**

The association between insurance and injury admission has not been examined.

**Methods:**

This was an observational retrospective cohort study of minor isolated extremity firearm injury captured in the Healthcare Cost and Utilization Project State Inpatient and Emergency Department Databases in 6 states (New York, Arkansas, Wisconsin, Massachusetts, Florida, and Maryland) from 2016 to 2017 among patients aged 16 years or older. The primary exposure was insurance. Admitted patients were propensity score matched to nonadmitted patients on age, extremity Abbreviated Injury Score, and Elixhauser Comorbidity Index with exact matching within hospital to adjust for selection bias. A general estimating equation logistic regression estimated the association between insurance and odds of admission in the matched cohort while controlling for sex, race, injury intent, injury type, hospital profit type, and trauma center designation with observations clustered by propensity score-matched pairs within hospital.

**Results:**

A total of 8151 patients presented to hospital with a minor isolated extremity firearm injury between 2016 and 2017 in 6 states. Patients were 88.0% male, 56.6% Black, and 71.7% aged 16 to 36 years old, and 22.1% were admitted. A total of 2090 patients were matched on propensity for admission. Privately insured matched patients had 1.70 higher adjusted odds of admission and 95% confidence interval of 1.30 to 2.22, compared with uninsured after adjusting for patient and hospital characteristics.

**Conclusions:**

Insurance was associated with hospital admission for minor isolated extremity firearm injury.

## Abstract 2

Mini Abstract:

This study sought to quantify the association between insurance and hospital admission following minor isolated extremity firearm injury. The main finding was that after matching for propensity to admit, privately insured patients had higher adjusted odds of admission compared with uninsured patients.

## INTRODUCTION

Patients with minor isolated extremity firearm injuries have historically been treated within the emergency department and discharged with “follow-up.”^1–3^ Most emergency departments have few resources to devote to outpatient care coordination of patients with minor injuries.^4,5^ Resources for home-based recovery from minor isolated extremity firearm injuries have been limited.^6^ Yet, even minor isolated extremity firearm injuries can lead to difficulties with activities of daily living at long-term follow-up.^7^

Clinicians can decide whether to admit patients with minor isolated extremity firearm injuries to hospital for inpatient pain control, physical therapy assessment, or comprehensive reassessment of the environment where they were injured. Inpatient hospital admission provides more time to coordinate postdischarge care, such as applying for health insurance, physical or occupational therapy, scheduling primary or wound follow-up care, arranging home health services, and evaluating social drivers of health.^8,9^ There is a literature gap evaluating patient characteristics associated with hospital admission for discretionary cases, such as minor isolated extremity firearm injuries.

The purpose of the study was to determine if health insurance was associated with inpatient hospital admission after minor isolated extremity firearm injuries. The first aim was to understand if between matched admitted and nonadmitted patients, privately insured individuals with minor isolated extremity firearm injuries would have greater adjusted odds of admission compared with uninsured individuals. The second aim was to compare discharge disposition and reinjury rates between matched admitted and nonadmitted patients. The hypothesis was that matched privately insured individuals with minor isolated extremity firearm injuries would have greater adjusted odds of admission compared with uninsured individuals and would have more frequent discharge to postacute care facilities and home assistance.

## METHODS

### Study Design

This was an observational retrospective cohort study evaluating the association between insurance and hospital admission among patients injured by a firearm from 2016 to 2017 across 6 states in the United States using data from the Healthcare Cost and Utilization Project (HCUP). This study was approved by the University Institutional Review Board. The reporting followed the strengthening the reporting of observational studies in epidemiology reporting guidelines.^10^

### Data Sources

Three data sets were used across New York, Arkansas, Wisconsin, Massachusetts, Florida, and Maryland to identify patients who presented to hospital after a minor isolated extremity firearm injury. The Statewide Inpatient Database (SID) and State Emergency Department Databases (SEDD) were developed by the HCUP.^11^ These data are statewide all-payer claims data sets that contain the inpatient and emergency department encounters for acute care hospitals. The combination of SID and SEDD includes all hospital visits for fatal and nonfatal injuries within the states above. The states above were included because they contained a patient identifier, “VisitLink,” which allowed linkage between all subsequent hospital encounters within the calendar year. These 6 states also provided hospital identifiers to link the SID and SEDD to the American Hospital Association (AHA) Survey data for detailed hospital characteristics.^12^

### Study Population

Encounters for adult patients (age ≥16) who presented to an emergency department with a minor isolated extremity injury were identified using the International Classification of Diseases and Related Health Problems (ICD), 10th Revision (Supplemental Table 1, see http://links.lww.com/AOSO/A333). ICD external causes of morbidity codes that delineated firearm injury were included. Patients’ initial encounters with minor isolated extremity injuries were identified with the indicator “A” at the end of the ICD code to indicate that this was the new injury (eg, W320XXA). Only minor injuries were included as defined by an Abbreviated Injury Scale (AIS) scores ≤2. Injury body region and severity were determined by converting ICD-10 injury diagnosis codes to AIS scores using the ICD Programs for Injury Categorization (ICDPIC).^13^ Injuries were categorized into different types (eg, fracture and dislocation) using the Center of Disease Control (CDC) Injury Diagnosis Matrix.^14^ Only encounters with isolated extremity injuries, defined as any patient with an upper or lower extremity AIS without coded injuries in other body regions, were included (Supplemental Figure 1, see http://links.lww.com/AOSO/A332).

### Primary Predictor

The primary predictor was insurance status. Insurance status was captured using the expected primary payer reported by hospitals upon discharge. Insurance status was categorized as Medicare, Medicaid, private insurance, uninsured, and other. Other included Worker’s Compensation, CHAMPUS, CHAMPVA, Title V, and other government programs.

### Primary Outcome

Hospital admission was determined based on whether the encounter was in the SID or only in SEDD. Emergency department discharges not admitted to hospital were defined as an SEDD encounter discharged from the emergency department without an accompanying SID record. Secondary outcomes considered included discharge disposition and reinjury. Discharge disposition was classified into 4 categories, home, transfer to acute care facility, transfer to postacute care facility/home health assistance, and other. Transfer to acute care facility was defined by the presence of SID encounter after SEDD encounter with corresponding admission and discharge dates, respectively, with identical VisitLink variable. Postacute care facility was defined as a discharge disposition of hospice, rehabilitation, long-term care hospital, psychiatric hospital, skilled nursing facility, or intermediate care facility and home health care. Other included leaving against medical advice and death. Reinjury was defined as a dichotomous variable based on whether a patient presented during the same year with a new firearm injury. The VisitLink variable was used to link subsequent hospital encounters to individual patients to estimate the rate of new firearm injuries after a minor isolated extremity firearm injury. Reinjury was classified as positive only if the patients had another later encounter with a firearm injury code with the ICD code indicator “A” in the same calendar year in SID or SEDD.

### Patient Characteristics

Patient demographic and injury characteristics hypothesized to be associated with discretionary inpatient hospital admission were age, race, median income quartile, injury intent, injury type, and injury severity. Age was categorized into 16 to 36 years, 37 to 64 years, and greater or equal to 65 years old. Race/ethnicity was reported as non-Hispanic White, non-Hispanic Black, Hispanic, and other, which included multiracial, self-described, native, Asian/Pacific Islander. Race/ethnicity was presented to characterize race as a social construct as opposed to race as a biological driver for disparity in admission practices. Median income quartile was determined through the patient’s zip code from HCUP linked to Claritas ZIP Code-demographic data. The quartiles for 2016 were defined as Q1 = $1 to $42,999, Q2 = $43,000 to $53,999, Q3 = $ 54,000 to $70,999, and Q4 ≥ $71,000. The quartiles for 2017 were Q1 = $1 to $43,999, Q2 = $44,000 to $55,999, Q3 = $56,000 to $73,999, and Q4 ≥ $74,000. Injury intent was classified as unintentional injury, self-inflicted, assault, undetermined, and legal intervention. Injury type was classified as open wound/superficial injury, fracture/dislocation, and other using the CDC Injury Diagnosis Matrix.^14^ Injury severity was an ordinal variable classified as Extremity AIS using ICDPIC.^13^ Patient comorbidities were calculated using the Weighted Elixhauser Index of Comorbidity.^15–18^

### Hospital Characteristics

Hospital characteristics hypothesized to be associated with inpatient hospital admission were bed size, teaching, medical school affiliation, urbanicity, trauma center designation, hospital profit status, and frequency of Medicaid discharges. Hospital bed size was collapsed into 4 categories <100, 100 to 299, 300 to 499, and ≥500 beds. Teaching status was captured by the American College of Graduate Medical Education accreditation classified as postgraduate teaching hospital or nonteaching hospital. Medical school affiliation was classified as medical school affiliated or nonaffiliated. Urbanicity was captured Core-Based Statistical Area classified as metro, micro, or rural. Trauma center designation was defined by AHA-specified trauma designation and when missing (13%) trauma designations were supplemented through state websites. Hospital profit status was classified as nonprofit, for profit, and government. Hospitals frequency of Medicaid discharges was defined based on all admissions for all causes by AHA Survey and classified as <10%, 10% to 19.9%, 20% to 49.9%, and ≥50%. Missing data were treated as missing at random and not imputed due to missingness of <1% in variables utilized for the analysis.

### Statistical Analysis

Patient and hospital characteristics were compared in the total initial unmatched admitted cohort, and nonadmitted cohort using mixed model bivariate logistic regression with admission as the outcome and the characteristic of interest as the lone fixed effect with patient ID as a random intercept. A multivariable generalized estimating equation logistic regression predicting hospital admission included insurance, age, race, sex, injury intent, injury type, weighted Elixhauser Comorbidity Index, trauma center designation, and hospital profit type with observations clustered by subject within hospital. Discharge disposition and reinjury were compared using bivariate logistic regression using subject-level random intercepts and admission status as the outcome.

Propensity score matching was employed to address the clinician selection bias in the admission. Based on the results of the above multivariable analysis, admitted and nonadmitted patients were matched on age, extremity AIS score, and Weighted Elixhauser Comorbidity Index. Exact matching within hospitals was specified, meaning that matched patient pairs were sampled from the same hospital to mitigate bias from hospital practice pattern differences. A 1:2 greedy match was used to preserve sample size with a caliper width of 0.2 to avoid dissimilar pairs. This caliper width was within a range that minimized the mean squared error by greater than 95%.^19^

Patient and hospital characteristics were compared in the matched admitted cohort versus nonadmitted cohort using mixed model logistic regression with admission status as the outcome and the characteristic of interest as the fixed effect with subject-level random intercepts. Informed by these results, a generalized estimating logistic regression model was used to evaluate the association between insurance status and inpatient hospital admission while controlling for sex, race/ethnicity, injury intent, injury type, hospital profit type, and trauma level designation with observations clustered by matched pair within hospital. Self-inflicted injured patients were collapsed into the “other” intent for the propensity-matched model because of the extremely small sample size (64 patients in 2 years across 6 states). The critical trend in intent we successfully preserved in the propensity matching model was the higher likelihood of assaults to be admitted and the lower likelihood of unintentional injuries to be discharged home. Collapsing self-inflicted ensured that we could run the propensity-matched analysis to control for selection bias in admission. Discharge disposition and reinjury were compared between matched admitted and nonadmitted patients with minor isolated firearm extremity injury using mixed model logistic regression with listed characteristics as the outcome and admission as the lone fixed effect and subject ID as the random intercept.

A sensitivity analysis was performed by identifying individuals who underwent procedures (Supplemental Table 2, see http://links.lww.com/AOSO/A334) to determine if admission rates differed. Bivariate analyses were performed to compare the admitted cohort versus the nonadmitted cohort among individuals who underwent procedures. Patient and hospital characteristics among the admitted and nonadmitted patients who underwent procedures were analyzed using generalized estimating logistic regression with admission as the outcome and race, injury type, Elixhauser Comorbidity score, intent, zip code quartile, and hospital bed size as the fixed effects. Observations were clustered by subject within hospital. Statistical analyses were performed using SAS 9.4 (Cary, NC). All *P* values were 2-tailed and *P* < 0.05 were considered significant.

## RESULTS

### Population Characteristics

A total of 8151 unique patients had a minor isolated extremity firearm injury with AIS 1 or 2 in New York, Arkansas, Wisconsin, Massachusetts, Florida, and Maryland from 2016 to 2017. Patients were 88.0% male, 56.6% Black, and 71.7% were between the ages of 16 and 36 years. Only 1800 (22.1%) were admitted. A total of 1110 (13.6%) patients were reinjured presenting to hospital with a new firearm injury.

In the overall unmatched cohort, there were significant differences in patient and hospital characteristics between admitted and nonadmitted patients. A smaller proportion of admitted patients, 28.8%, had an AIS score of 2 compared with 33.3% of nonadmitted patients, *P* < 0.001 (Supplemental Table 3, see http://links.lww.com/AOSO/A335). Admitted patients had a higher mean Elixhauser Comorbidity Index of 0.9, SD 1.25, compared with 0.3, SD 0.62, among nonadmitted patients, *P* < 0.001. Patients in large, teaching, metropolitan level 1 trauma centers that were for-profit hospitals were most likely to be admitted (Supplemental Table 4, see http://links.lww.com/AOSO/A336). A larger proportion of admitted patients, 13.6%, were discharged to postacute care facilities or home health care compared with 1.6% among nonadmitted patients, *P* < 0.001 (Supplemental Table 5, see http://links.lww.com/AOSO/A337). Patient age, injury, intent, comorbidities, and hospital characteristics were associated with the highest adjusted odds of admission (Supplemental Table 6, see http://links.lww.com/AOSO/A338).

### Propensity-Matched Cohort Characteristics

There were 2090 (26%) patients matched on propensity for admission. The matched cohort was better balanced though some differences between admitted and nonadmitted patients persisted. A larger proportion of admitted patients, 47.3%, were survivors of an assault, compared with 37.6% of nonadmitted patients, *P* < 0.001 (Table 1). A larger proportion of admitted patients, 55.6%, had a fracture or dislocation compared with 45.0% among nonadmitted patients, *P* < 0.001. The hospital differences were mostly eliminated with matching with the exception that metropolitan hospitals accounted for a slightly lower proportion of patients admitted, 95.9%, compared with nonadmitted, 97.7%, *P* = 0.02, but the difference was statistically but not clinically significant (Table 2).

**TABLE 1.** Patient Characteristics of Propensity Score-Matched Admitted Versus Nonadmitted Patients With a Minor Isolated Extremity Firearm Injury Presenting to Hospitals in New York, Arkansas, Wisconsin, Massachusetts, Florida, and Maryland From 2016 to 2017 (N = 2090)

	Not Admitted	Admitted	
Variables	n = 1230, No. (%)	n = 860, No. (%)	*P* *
Age, y			
16 to 36	932 (75.8)	651 (75.7)	0.13
37 to 64	247 (20.1)	187 (21.7)	
>65	51 (4.1)	22 (2.6)	
Sex			
Male	1084 (88.1)	748 (87.0)	0.44
Female	146 (11.9)	112 (13.0)	
Race/ethnicity			
White (NH)	304 (24.9)	219 (25.8)	0.70
Black or African American (NH)	767 (62.9)	515 (60.6)	
Hispanic	110 (9.0)	84 (9.9)	
Other (NH)†	39 (3.2)	32 (3.8)	
Insurance			
Uninsured	417 (34.0)	213 (24.8)	
Medicaid	418 (34.1)	349 (40.6)	<0.001
Medicare	237 (19.3)	194 (22.6)	
Private	74 (6.0)	41 (4.8)	
Other‡	80 (6.5)	63 (7.3)	
Zip code income quartile§			
1	681 (56.0)	489 (57.7)	0.84
2	265 (21.8)	172 (20.3)	
3	178 (14.7)	126 (14.9)	
4	91 (7.5)	61 (7.2)	
Injury intent			
Assault	463 (37.6)	407 (47.3)	<0.001
Unintentional	712 (57.9)	413 (48.0)	
Other‖	55 (4.5)	40 (4.7)	
Injury type			
Fracture/dislocation	553 (45.0)	478 (55.6)	<0.001
Wound/superficial injury	654 (53.2)	368 (42.8)	
Other	23 (1.9)	14 (1.6)	
Extremity Abbreviated Injury Scale (AIS)			
1	855 (69.5)	589 (68.5)	0.62
2	375 (30.5)	271 (31.5)	
Elixhauser Comorbidity Index, mean (SD)	0.4 (0.76)	0.4 (0.74)	0.41

* Mixed effects logistic regression with variable as fixed effect and subject ID as random effect.

† Other included multiracial, self-described, native, Asian/Pacific Islander.

‡ Other insurance included Worker’s Compensation, CHAMPUS, CHAMPVA, Title V, and other government.

§ Zip income quartile classification was median household income of residents in the patient’s zip code.

‖ Other included legal intervention, self-inflicted, and undetermined.

NH indicates non-Hispanic.

**TABLE 2.** Hospital Characteristics for Propensity Score-Matched Admitted Versus Nonadmitted Patients With a Minor Isolated Extremity Firearm Injury Presenting to Hospitals in New York, Arkansas, Wisconsin, Massachusetts, Florida, and Maryland From 2016 to 2017 (N = 2090)

	Not Admitted	Admitted	
	n = 1230, No. (%)	n = 860, No. (%)	*P* *
Hospital bed size			
<100	24 (2.0)	28 (3.3)	0.17
100 to 299	194 (15.8)	150 (17.5)	
300 to 499	310 (25.2)	220 (25.6)	
≥500	701 (57.0)	461 (53.7)	
Hospital teaching status			
Teaching	1001 (81.4)	692 (80.6)	0.61
Nonteaching	228 (18.6)	167 (19.4)	
Medical school affiliated			
Affiliated	888 (72.3)	615 (71.6)	0.74
Nonaffiliated	341 (27.7)	244 (28.4)	
CBSA type			
Metro	1201 (97.7)	824 (95.9)	0.02
Micro	23 (1.9)	20 (2.3)	
Rural	5 (0.4)	15 (1.7)	
Trauma center level			
Nontrauma	154 (12.6)	132 (15.4)	0.29
Level 1	602 (49.3)	408 (47.7)	
Level 2	404 (33.1)	268 (31.3)	
Level 3+	62 (5.1)	48 (5.6)	
Hospital profit			
Nonprofit	749 (60.9)	523 (60.9)	0.98
For profit	224 (18.2)	159 (18.5)	
Government	256 (20.8)	177 (20.6)	
Percent Medicaid discharges			
<10%	40 (3.3)	31 (3.6)	0.90
10% to 19.9%	258 (21.0)	188 (21.9)	
20% to 49.9%	797 (64.8)	544 (63.3)	
≥50%	134 (10.9)	96 (11.2)	

* Calculated using mixed model logistic regression with admission as the outcome and the listed characteristic as the lone fixed effect and subject ID as the random intercept.

CBSA indicates Core-Based Statistical Area.

Upon multivariable analysis of the matched cohort, unintentional injuries had a 0.58 lower adjusted odds of being admitted than assault-injured patients, with 95% confidence interval of 0.48 to 0.71(Table 3). Fracture/dislocations injuries had a 1.53 increased adjusted odds of being admitted compared with those with wounds/superficial injuries, with 95% confidence interval of 1.27 to 1.84. Privately insured matched patients had a 1.70 higher adjusted odds of admission, with 95% confidence interval of 1.29 to 2.22, compared with uninsured after adjusting for patient and hospital characteristics.

**TABLE 3.** Association Between Insurance Status and Admission Status Using Propensity Score-Matched Admitted Versus Nonadmitted Patients With a Minor Isolated Extremity Firearm Injury Presenting to Hospitals in New York, Arkansas, Wisconsin, Massachusetts, Florida, and Maryland From 2016 to 2017 (N = 2090)

	Odds Ratio*	95% Confidence Interval*
Sex		
Male	Ref	—
Female	1.14	0.87 to 1.49
Race/ethnicity		
White (NH)	Ref	—
Black or African American (NH)	0.84	0.66 to 1.06
Hispanic	0.98	0.69 to 1.34
Other (NH)†	1.14	0.68 to 1.92
Insurance		
Uninsured	Ref	—
Private	1.70	1.29 to 2.22
Medicare	0.69	0.44 to 1.07
Medicaid	1.00	0.77 to 1.29
Other‡	0.99	0.67 to 1.45
Injury intent		
Assault	Ref	—
Unintentional	0.58	0.48 to 0.71
Other§	0.84	0.55 to 1.30
Injury type		
Wound/superficial injury	Ref	—
Fracture/dislocation	1.53	1.27 to 1.84
Other	0.96	0.47 to 1.97
Trauma center level		
Nontrauma	Ref	—
Level 1	0.67	0.49 to 0.91
Level 2	0.76	0.57 to 1.02
Level 3+	0.82	0.50 to 1.32
Hospital profit		
Nonprofit	Ref	—
For profit	1.16	0.88 to 1.51
Government	1.14	0.90 to 1.44

* Generated from a hierarchical multivariable logistic regression model used to evaluate the association between insurance status and inpatient hospital admission while controlling for sex, race/ethnicity, injury intent, injury type, hospital profit type, and trauma level designation accounting for clustering at the hospital and matched-pair level.

† Other included multiracial, self-described, native, Asian/Pacific Islander.

‡ Other included Worker’s Compensation, CHAMPUS, CHAMPVA, Title V, and other government programs.

§ Other included legal intervention, self-inflicted, and undetermined.

NH indicates non-Hispanic; Ref, reference.

Matched admitted patients were more likely to be discharged to postacute care facilities or home health care, 11.3%, compared with 1.7% among nonadmitted patients, *P* < 0.001 (Table 4). Reinjury was similar between admitted and nonadmitted matched patients.

**TABLE 4.** Discharge Disposition and Reinjury of Propensity Score-Matched Admitted Versus Nonadmitted Patients With a Minor Isolated Extremity Firearm Injury Presenting to Hospitals in New York, Arkansas, Wisconsin, Massachusetts, Florida, and Maryland From 2016 to 2017 (N = 2090)

Outcomes	Not Admitted	Admitted	*P* *
	n = 1230, No. (%)	n = 860, No. (%)	
Discharge disposition†			
Home	1129 (91.8)	667 (77.6)	<0.001
Transfer to acute care facility	57 (4.6)	75 (8.7)	
Postacute care facility‡/home health care	20 (1.6)	97 (11.3)	
Against medical advice	24 (2.0)	21 (2.4)	
Reinjury§	168 (13.7)	110 (12.8)	0.57

* *P* values calculated using mixed model logistic regression with listed characteristic as the outcome and admission as the fixed effect and subject ID as the random intercept.

† Categories consolidated to facilitate model convergence in cases of sparse cell sizes.

‡ Postacute care includes discharge to hospice, rehabilitation, long-term care hospital, psychiatric hospital, skilled nursing facility, or intermediate care facility.

§ Reinjury was defined as whether or not a patient presented to any hospital during the same calendar year for a new firearm injury.

Sensitivity analyses examining only those in the unmatched cohort who received procedures were limited by a skewed distribution toward admitted patients (n = 741 of 770, 94.6%). Most frequent procedures were blood transfusion (Supplemental Table 7, see http://links.lww.com/AOSO/A339). No significant differences were found between the admitted and nonadmitted patients based on whether they received a procedure in age, sex, race/ethnicity, insurance status, zip code income quartile, intent, and AIS score (Supplemental Table 8, see http://links.lww.com/AOSO/A340). Similar to the larger cohort, however, a significantly higher number of the admitted receiving procedures experienced fracture/dislocation injuries (79.2%) compared with the nonadmitted (53.7%, *P* = 0.02). Those who were admitted also had greater comorbidity than the nonadmitted (mean Elixhauser Comorbidity Index = 1.0 vs 0.4, *P* = 0.04). There were no differences in hospital characteristics (Supplemental Table 9, see http://links.lww.com/AOSO/A341). Upon multivariable analysis, there was no difference in insurance status among individuals who underwent procedures and were admitted compared with nonadmitted individuals (Supplemental Table 10, see http://links.lww.com/AOSO/A342).

## DISCUSSION

This study sought to determine if insurance status was associated with inpatient hospital admission after minor isolated extremity firearm injuries. Between matched admitted and nonadmitted patients, privately insured patients with minor isolated extremity firearm injuries had greater adjusted odds of admission compared with uninsured patients after adjusting for patient and hospital characteristics. Admitted patients in the propensity-matched cohort were more frequently discharged to another acute care, postacute care facility, or with home health care.

Various social drivers of health, including insurance status, may indirectly influence the shared decision with the patient and may also be impacted by admission itself. Health insurance is associated with both appropriate and inappropriate health care use.^20,21^ A self-pay patient may be more likely to wish not to be admitted because they bear the full burden of the health care cost. Furthermore, it is important that the insurance status listed for each patient is *the insurance that is billed upon discharge*. Many of our firearm-injured patients are uninsured upon admission and our multidisciplinary teams work quite hard to ensure they become insured upon discharge. This ensures hospitals will be remunerated for their inpatient care. It is likely that admitting a patient enables the social workers to apply for insurance as well as other outpatient support services such as home health as we observed. Emergency department social workers do not have the bandwidth or time to perform such activities in the few hours they interact with patients. Health insurance access can improve the timeliness of diagnosis, improve in-hospital and long-term survival, and increase overall health care utilization.^22–26^ Not all firearm injuries necessitate inpatient admission for direct medical care.^1^ Nguyen et al^27^ found that costs of care were reduced for patients with extremity firearm injuries who were treated with antibiotics and discharged from the emergency department. These cost savings must be weighed against the long-term cost associated with reinjury-associated readmission, which Spitzer et al^28^ found that 10% of total costs of firearm injury were due to readmission within the first 6 months after injury.

The implication of the study findings should be taken in the context of the lack of social safety net and social isolation in the United States, which can mean patients with minor isolated injuries do not have any support mechanisms in their community. Complications of isolated extremity injuries include vascular injury, nerve injury, and wound infections.^29^ Firearm-injured individuals are known to have new functional limitations, daily pain, and poor physical health-related quality of life.^30,31^ As a result, some hospitals have developed specific admission criteria for medical or social conditions.^32,33^ However, the medical community should reconsider whether there is an alternative to supporting patients with minor isolated extremity firearm injuries beyond inpatient admission. Examples can be outpatient transitional care programs, which can be cost-saving and reduce hospital use.^34^ Community-based organizations can address many of the social drivers of health that put patients at risk for injury and illness. Learning to effectively partner with these organizations will be critical given the new Center for Medicare and Medicaid Services Framework for Health Equity that since January 2024 requires hospitals to collect, and soon address, social drivers of health in their communities.

### Limitations

This study has several limitations. First, these all-payer claims data provide universal capture of all acute care hospital emergency department and inpatient encounters but provide very limited clinical granularity. For example, presenting symptoms such as vital signs were not captured in this data set. This bias was addressed by reducing sample heterogeneity of injury severity and focusing on isolated extremity firearm injuries with an AIS cutoff of 2 or less. Second, the standard mechanism to estimate AIS, ICDPIC, may have underestimated the AIS.^35,36^ This bias was addressed by also classifying injury type using ICD codes and the CDC Injury Matrix, then excluding patients with burns, crush, and nonisolated injuries as well as controlling for injury type in all multivariable regressions. Third, the reinjury secondary outcome was limited by the fact that the VisitLink variable was assigned by calendar year. The study underestimated the reinjury rates of patients within this data set. However, evidence suggests that most reinjuries occur within the first year.^37^ Fourth, the VisitLink variable was missing for 14% of the firearm-injured cohort. The VisitLink variable was created using identifiable patient data missing among undocumented and undomiciled patients. This likely led to the exclusion of undocumented and undomiciled patients from this study causing sampling bias. Sampling bias again likely underestimated reinjury rates since undomiciled have higher rates of assault,^38^ though presumably the underestimation of reinjury rates would have been similarly distributed between admitted and nonadmitted patients therefore not impacting the main study findings. Finally, only 6 states were included in the study due to the limited availability of VisitLink and AHA ID across states contributing to SID and SEDD leading to sampling bias as well. Nonetheless, geographically, demographically, and politically diverse states were selected to maximize generalizability.

## CONCLUSIONS

This observational retrospective cohort study of minor firearm injuries across 6 states found that privately insured patients with minor isolated extremity firearm injuries had greater adjusted odds of admission compared with uninsured patients after adjusting for patient and hospital characteristics. Matched admitted patients were more frequently discharged to another acute care or postacute care facility or with home health assistance compared with nonadmitted patients. Reinjury rates were not different between matched admitted and nonadmitted patients.

## ACKNOWLEDGMENTS

A.C.T. and A.M.S. had full access to all the data in the study and take responsibility for the integrity of the data and the accuracy of the data analysis.

## Supplementary Material























## References

[1] Ordog GJ Wasserberger J Balasubramanium S. Civilian gunshot wounds-outpatient management. J Trauma. 1994;36:106–111. 8295233

[2] Jabara JT Gannon NP Vallier HA. Management of civilian low-velocity gunshot injuries to an extremity. J Bone Joint Surg Am. 2021;103:1026–1037. 33755646 10.2106/JBJS.20.01544

[3] Gani F Sakran JV Canner JK. Emergency department visits for firearm-related injuries in the United States, 2006-14. Health Aff (Millwood). 2017;36:1729–1738. 28971917 10.1377/hlthaff.2017.0625

[4] Primm K Muraleetharan D Gilreath T. Use of emergency departments for preventative care among adults in the United States: estimates from the 2017 National Health Interview Survey. J Emerg Med. 2019;57:578–586. 31477312 10.1016/j.jemermed.2019.06.004

[5] Gordon JA. The hospital emergency department as a social welfare institution. Ann Emerg Med. 1999;33:321–325. 10036347 10.1016/s0196-0644(99)70369-0

[6] Dalve K Gause E Mills B. Neighborhood disadvantage and firearm injury: does shooting location matter? Inj Epidemiol. 2021;8:10. 33678193 10.1186/s40621-021-00304-2 PMC7938602

[7] Abghari M Monroy A Schubl S. Outcomes following low-energy civilian gunshot wound trauma to the lower extremities: results of a standard protocol at an urban trauma center. Iowa Orthop J. 2015;35:65–69. 26361447 PMC4492129

[8] Purtle J Dicker R Cooper C. Hospital-based violence intervention programs save lives and money. J Trauma Acute Care Surg. 2013;75:331–333. 23887566 10.1097/TA.0b013e318294f518

[9] Purtle J Rich LJ Bloom SL. Cost-benefit analysis simulation of a hospital-based violence intervention program. Am J Prev Med. 2015;48:162–169. 25442223 10.1016/j.amepre.2014.08.030

[10] von Elm E Altman DG Egger M; STROBE Initiative. Strengthening the Reporting of Observational Studies in Epidemiology (STROBE) statement: guidelines for reporting observational studies. BMJ. 2007;335:806–808. 17947786 10.1136/bmj.39335.541782.AD PMC2034723

[11] Healthcare Cost and Utilization Project (HCUP) Statistical Briefs. Agency for Healthcare Research and Quality; 2006. 21413206

[12] Kralovec PD Mullner R. The American hospital association’s annual survey of hospitals: continuity and change. Health Serv Res. 1981;16:351–355. 7298343 PMC1072253

[13] Clark DE Black AW Skavdahl DH. Open-access programs for injury categorization using ICD-9 or ICD-10. Inj Epidemiol. 2018;5:11. 29629480 10.1186/s40621-018-0149-8 PMC5890002

[14] Hedegaard H Johnson RL Garnett MF. The 2020 International Classification of Diseases, 10th revision, clinical modification injury diagnosis framework for categorizing injuries by body region and nature of injury. Natl Health Stat Report. 2020;150:1–27. 33395385

[15] Shaka H Edigin E. A revised comorbidity model for administrative databases using clinical classifications software refined variables. Cureus. 2021;13:e20407. 35047250 10.7759/cureus.20407 PMC8756739

[16] Moore BJ White S Washington R. Identifying increased risk of readmission and in-hospital mortality using hospital administrative data: the AHRQ Elixhauser Comorbidity Index. Med Care. 2017;55:698–705. 28498196 10.1097/MLR.0000000000000735

[17] Menendez ME Ring D. A comparison of the Charlson and Elixhauser Comorbidity measures to predict inpatient mortality after proximal humerus fracture. J Orthop Trauma. 2015;29:488–493. 26165266 10.1097/BOT.0000000000000380

[18] Elixhauser A Steiner C Harris DR. Comorbidity measures for use with administrative data. Med Care. 1998;36:8–27. 9431328 10.1097/00005650-199801000-00004

[19] Austin PC. Optimal caliper widths for propensity-score matching when estimating differences in means and differences in proportions in observational studies. Pharm Stat. 2011;10:150–161. 20925139 10.1002/pst.433 PMC3120982

[20] Lohr KN Brook RH Kamberg CJ. Use of medical care in the Rand Health Insurance Experiment. Diagnosis- and service-specific analyses in a randomized controlled trial. Med Care. 1986;24(9 Suppl):S1–87. 3093785

[21] Brook RH Appel FA. Quality-of-care assessment: choosing a method for peer review. N Engl J Med. 1973;288:1323–1329. 4706275 10.1056/NEJM197306212882504

[22] Zhao J Han X Nogueira L. Health insurance status and cancer stage at diagnosis and survival in the United States. CA Cancer J Clin. 2022;72:542–560. 35829644 10.3322/caac.21732

[23] Pancholy SB Patel GA Patel DD. Association between insurance status and in-hospital outcomes in patients with out-of-hospital ventricular fibrillation arrest. Clin Cardiol. 2021;44:511–517. 33660870 10.1002/clc.23564 PMC8027577

[24] Rogers MAM Lee JM Tipirneni R. Interruptions in private health insurance and outcomes in adults with type 1 diabetes: a longitudinal study. Health Aff (Millwood). 2018;37:1024–1032. 29985705 10.1377/hlthaff.2018.0204 PMC6590517

[25] Baicker K Taubman SL Allen HL; Oregon Health Study Group. The Oregon experiment-effects of Medicaid on clinical outcomes. N Engl J Med. 2013;368:1713–1722. 23635051 10.1056/NEJMsa1212321 PMC3701298

[26] Baicker K Allen HL Wright BJ. The effect of Medicaid on management of depression: evidence from the Oregon Health Insurance Experiment. Milbank Q. 2018;96:29–56. 29504203 10.1111/1468-0009.12311 PMC5835676

[27] Nguyen MP Savakus JC Reich MS. Costs of care for low-energy extremity gunshot injuries are reduced with standardized treatment. J Orthop Trauma. 2021;35:e61–e63. 32569067 10.1097/BOT.0000000000001870

[28] Spitzer SA Vail D Tennakoon L. Readmission risk and costs of firearm injuries in the United States, 2010-2015. PLoS One. 2019;14:e0209896. 30677032 10.1371/journal.pone.0209896 PMC6345420

[29] Berg RJ Okoye O Inaba K. Extremity firearm trauma: the impact of injury pattern on clinical outcomes. Am Surg. 2012;78:1383–1387. 23265128

[30] Herrera-Escobar JP de Jager E McCarty JC. Patient-reported outcomes at 6 to 12 months among survivors of firearm injury in the United States. Ann Surg. 2021;274:e1247–e1251. 32530586 10.1097/SLA.0000000000003797

[31] Herrera-Escobar JP deRoon-Cassini T Brasel K. Development and validation of a revised trauma-specific quality of life instrument. J Trauma Acute Care Surg. 2020;88:501–507. 31626032 10.1097/TA.0000000000002505

[32] Havens PL Butler JC Day SE. Treating measles: the appropriateness of admission to a Wisconsin children’s hospital. Am J Public Health. 1993;83:379–384. 8438976 10.2105/ajph.83.3.379 PMC1694644

[33] MacDonell R Woods O Whelan S. Interventions to standardise hospital care at presentation, admission or discharge or to reduce unnecessary admissions or readmissions for patients with acute exacerbation of chronic obstructive pulmonary disease: a scoping review. BMJ Open Respir Res. 2020;7:e000733. 10.1136/bmjresp-2020-000733 PMC7709517 33262103

[34] Shilati FM Silver CM Baskaran A. Transitional care programs for trauma patients: a scoping review. Surgery. 2023;174:1001–1007. 37550166 10.1016/j.surg.2023.06.038 PMC10527729

[35] Eskesen TO Sillesen M Rasmussen LS. Agreement between standard and ICD-10-based injury severity scores. Clin Epidemiol. 2022;14:201–210. 35221725 10.2147/CLEP.S344302 PMC8864409

[36] Wan V Reddy S Thomas A. How does injury severity score derived from ICDPIC Utilizing ICD-10-CM codes perform compared to injury severity score derived from TQIP? J Trauma Acute Care Surg. 2022;94:141–147. 35647796 10.1097/TA.0000000000003656 PMC9708941

[37] Sims DW Bivins BA Obeid FN. Urban trauma: a chronic recurrent disease. J Trauma. 1989;29:940–946; discussion 946. 2746704

[38] Silver CM Thomas AC Reddy S. Injury patterns and hospital admission after trauma among people experiencing homelessness. JAMA Netw Open. 2023;6:e2320862. 37382955 10.1001/jamanetworkopen.2023.20862 PMC10311388

