# Supplementary material for: Patient and Hospital Characteristics Associated with Admission Among Patients With Minor Isolated Extremity Firearm Injuries: A Propensity-Matched Analysis
Source: Ann Surg Open. 2024 May 6;5(2):e430. doi: 10.1097/AS9.0000000000000430 (PMC11191909; doi:10.1097/AS9.0000000000000430)
Supplement: Supplementary file 1 [file as9-5-e430-s001.pdf]

Supplemental Table 1. All firearm injured patients were isolated using International Statistical Classification of Diseases and Related Health Problems, Tenth Revision

"W320XXA","W321XXA","W3300XA","W3301XA","W3302XA","W3303XA","W3309XA","W3310XA","W3311XA","W3312XA","W3313XA","W3319XA","W3400XA","W3409XA","W3410XA","W3419XA"
"X72XXXA","X730XXA","X731XXA","X732XXA","X738XXA","X739XXA","X748XXA","X749XXA","X93XXXA","X940XXA","X941XXA","X942XXA","X948XXA","X949XXA","X958XXA","X959XXA"
"Y22XXXA","Y230XXA","Y231XXA","Y232XXA","Y233XXA","Y238XXA","Y239XXA","Y248XXA","Y249XXA","Y35001A","Y35002A","Y35003A","Y35009A","Y35011A","Y35012A","Y35013A","Y35019A","Y35021A","Y35022A","Y35023A","Y35029A","Y35031A","Y35032A","Y35033A","Y35039A","Y35091A","Y35092A","Y35093A","Y35099A"
