# Supplementary material for: Patient and Hospital Characteristics Associated with Admission Among Patients With Minor Isolated Extremity Firearm Injuries: A Propensity-Matched Analysis
Source: Ann Surg Open. 2024 May 6;5(2):e430. doi: 10.1097/AS9.0000000000000430 (PMC11191909; doi:10.1097/AS9.0000000000000430)
Supplement: Supplementary file 3 [file as9-5-e430-s003.pdf]

**Supplemental Table 2: Procedure Codes Used to Identify Procedures Among Minor Isolated Extremity Firearm Injuries**
Value $longbonefx 820.0,
820.00,
820.01,
820.02,
820.03,
820.09,
820.1,
820.10,
820.11,
820.12,
820.13,
820.19,
820.2,
820.21,
820.22,
820.3,
820.30,
820.31,
820.32,
820.8,
820.9,
821.0,
821.00,
821.01,
821.2,
821.20,
821.21,
821.22,
821.23,
821.29,
823.0,
823.00,
823.01,
823.02,
823.2,
823.20,
823.21,
823.22,
823.4,
823.40,
823.41,
823.42,
823.9,
823.90,

823.91,
823.92,
827.0
= 1
other = 0;
value $openfx 821.1,
821.10,
821.11,
821.3,
821.30,
821.31,
821.32,
821.33,
821.39,
823.1,
823.10,
823.11,
823.12,
823.3,
823.30,
823.31,
823.32,
823.8,
823.80,
823.81,
823.82,
812.1,
812.10,
812.11,
812.12,
812.13,
812.19,
812.3,
812.30,
812.31,
812.5,
812.50,
812.51,
812.52,
812.53,
812.54,
812.59,
813.1,
813.10,
813.11,
813.12,

813.13,
813.14,
813.15,
813.16,
813.17,
813.18,
813.3,
813.30,
813.31,
813.32,
813.33,
813.5,
813.50,
813.51,
813.52,
813.53,
813.54,
813.9,
813.90,
813.91,
813.92,
813.93,
828.1,
827.1,
829.1,
818.1
= 1
other = 0;
Value $extcrush 928.0,
928.00,
928.01,
928.1,
928.10,
928.8,
928.9,
927.0,
927.00,
927.01,
927.02,
927.03,
927.09,
927.1,
927.10,
927.8,
927.9,
929.0,

929.9
= 1
other = 0 ;
value $partialhip 81.51,
81.52
= 1
other = 0;
value $fasciotomy 83.14
= 1
other = 0;
value $ORIFLE 79.35,
79.36,
79.37,
79.38,
79.39
= 1
other = 0;
value $exfix 78.1,
78.10,
78.11,
78.12,
78.13,
78.14,
78.15,
78.16,
78.17,
78.18,
78.19
= 1
other = 0;
value $openfxdeb 79.6,
79.60,
79.61,
79.62,
79.63,
79.64,
79.65,
79.66,
79.67,
79.68,
79.69
= 1
other = 0;
value $Thrombemb 38.0, /*Incision of vessel
thrombectomy/embolectomy*/

38.00,
38.01,
38.02,
38.03,
38.04,
38.05,
38.06,
38.07,
38.08,
38.09
= 1
other = 0;
value $excisves 38.6, /*Excision of vessel*/
38.60,
38.61,
38.62,
38.63,
38.64,
38.65,
38.66,
38.67,
38.68,
38.69,
38.3,
/*Resection of vessel of Anastomosis*/
38.30,
38.31,
38.32,
38.33,
38.34,
38.35,
38.36,
38.37,
38.38,
38.39,
38.4,
38.40,
/*Resection of vessel with replacement*/
38.41,
38.42,
38.43,
38.44,
38.45,
38.46,
38.47,
38.48,

38.49,
38.21
= 1
other = 0;
Value $Endarterect 38.1, /*Endarterectomy*/
38.10,
38.11,
38.12,
38.13,
38.14,
38.15,
38.16,
38.17,
38.18,
38.19
= 1
other = 0;
Value $endovascrep 39.7, /*Endovascular Vascular
Repair*/
39.71,
39.72,
39.73,
39.74,
39.77,
39.79
= 1
other = 0;
38.86, /*Abdominal
arterial occlusion*/
38.87, /*Abdominal
arterial occlusion*/
38.88,
38.89 /*LE arterial
occlusion*/
= 1
other = 0 ;
value $vesselsut 39.3,
39.30,
39.31, /*suture of
artery*/
39.32 /*suture
of vein*/
= 1
other = 0 ;
value $vascrep 39.2, /*Bypass graft*/

39.20,
39.21,
39.22,
39.23,
39.24,
39.25,
39.26,
39.27,
39.28,
39.29,
39.5,
39.52,
39.53,
39.56,
39.57,
39.58, /*repair
of vessel with graft*/
39.59 /*vascular
repair*/
= 1
other = 0 ;
value $hemor_c 39.9,
39.91,
39.98,
39.99 /*control of
hemorrhage*/
= 1
other = 0 ;
value $skindebr 86,
86.0,
86.04, /*incision and drainage*/
86.05, /*Incision with removal of foreign body*/
86.09, /*Other incision and drainage*/
86.1,
86.10,
86.11,
86.19,
86.2,
86.20,
86.22, /*Excisional
debridement*/
value $embo_c 39.79, /*endovascular repair of vessel*/
99.29, /*prophylactic
emboli*/
88.47 /* Intra-abdominal angiography*/

= 1
other = 0 ;
value $ffp_c 99.07 /*ffp transfusion*/
= 1
other = 0 ;
value $plt_c 99.05 /*plt transfusion*/
= 1
other = 0 ;
value $prbc_c 99.04 /*prbc transfusion*/

= 1
