# Supplementary material for: Patient and Hospital Characteristics Associated with Admission Among Patients With Minor Isolated Extremity Firearm Injuries: A Propensity-Matched Analysis
Source: Ann Surg Open. 2024 May 6;5(2):e430. doi: 10.1097/AS9.0000000000000430 (PMC11191909; doi:10.1097/AS9.0000000000000430)
Supplement: Supplementary file 4 [file as9-5-e430-s004.pdf]

Supplemental Table 3. Patient Characteristics of Unmatched Admitted versus Non-admitted Patients with a Minor Isolated Extremity Firearm Injury Presenting to Hospitals in New York, Arkansas, Wisconsin, Massachusetts, Florida, and Maryland from 2016-2017 (N=8,151)

	**Not Admitted N=6,351** No (%)	**Admitted N=1,800** No (%)	**P-value[a]**
**Age (Years)**			
16-36	4551 (71.7)	1295 (71.9)	
37-64	1517 (23.9)	422 (23.4)	0.90
>65	283 (4.5)	83 (4.6)	
**Sex**			
Male	5585 (87.9)	1593 (88.5)	0.52
Female	766 (12.1)	207 (11.5)	
**Race/Ethnicity**			
White (NH)	1862 (29.5)	501 (28.1)	
Black or African American (NH)	3625 (57.5)	1012 (56.9)	0.16
Hispanic	590 (9.4)	190 (10.7)	
Other (NH)[b]	229 (3.6)	77 (4.3)	
**Insurance**			
Medicaid	2243 (35.5)	739 (41.1)	
Medicare	395 (6.2)	129 (7.2)	
Private	1402 (22.2)	376 (20.9)	<0.001
Uninsured	1933 (30.6)	415 (23.1)	
Other[c]	348 (5.5)	138 (7.7)	
**Zip Code Income Quartile[d]**			
1	3374 (54.0)	959 (54.1)	
2	1426 (22.8)	402 (22.7)	0.99
3	950 (15.2)	268 (15.1)	
4	499 (8.0)	145 (8.2)	
**Intent**			
Assault	2058 (32.4)	839 (46.6)	
Self-Inflicted	25 (0.4)	39 (2.2)	
Unintentional	4018 (63.3)	852 (47.3)	<0.001
Undetermined	205 (3.2)	59 (3.3)	
Legal Intervention	45 (0.7)	11 (0.6)	
**Injury Type**			
Fracture / Dislocation	934 (15.2)	1202 (70.4)	
Wound / Superficial Injury	5101 (82.8)	478 (28.0)	<0.001
Other	126 (2.0)	27 (1.6)	
**Extremity Abbreviated Injury Scale (AIS)**			
1	4203 (66.2)	1282 (71.2)	<0.001

2	2148 (33.8)	518 (28.8)	
**Elixhauser Comorbidity Score, Mean (SD)**	0.3 (0.62)	0.9 (1.25)	<0.001

NH=Non-Hispanic

a. Generated from mixed model univariate logistic regression with admission as the outcome and the listed characteristic as the lone fixed effect with patient ID as a random intercept.

b. Other included Multiracial, self-described, Native, Asian/Pacific-Islander

c. Other Insurance included Worker's Compensation, CHAMPUS, CHAMPVA, Title V, and other government programs.

d. Zip income quartile was a quartile classification of the estimated median household income of residents in the patient's ZIP Code.
