# Supplementary material for: Patient and Hospital Characteristics Associated with Admission Among Patients With Minor Isolated Extremity Firearm Injuries: A Propensity-Matched Analysis
Source: Ann Surg Open. 2024 May 6;5(2):e430. doi: 10.1097/AS9.0000000000000430 (PMC11191909; doi:10.1097/AS9.0000000000000430)
Supplement: Supplementary file 5 [file as9-5-e430-s005.pdf]

Supplemental Table 4: Hospital Characteristics for Unmatched Admitted versus Non-admitted Patients with a Minor Isolated Extremity Firearm Injury Presenting to Hospitals in New York, Arkansas, Wisconsin, Massachusetts, Florida, and Maryland from 2016-2017 (N=8,151)

	Not Admitted N=6,351 No. (%)	Admitted N=1,800 No. (%)	P-value[a]
**Hospital Bed Size**			
<100	550 (8.7)	53 (3.0)	<0.001
100-299	1590 (25.1)	347 (19.5)	
300-499	1460 (23.0)	512 (28.8)	
≥500	2735 (43.2)	866 (48.7)	
**Hospital Teaching Status**			
Teaching	4538 (71.6)	1369 (77.0)	<0.001
Non-Teaching	1797 (28.4)	409 (23.0)	
**Medical School Affiliated**			
Affiliated	3946 (62.3)	1217 (68.4)	<0.001
Non-Affiliated	2389 (37.7)	561 (31.6)	
**CBSA Type**			
Metro	5873 (92.7)	1709 (96.1)	<0.001
Micro	275 (4.3)	53 (3.0)	
Rural	187 (3.0)	16 (0.9)	
**Trauma Center Level**			
Non-Trauma	1814 (28.8)	313 (17.7)	<0.001
Level 1	2198 (34.8)	798 (45.0)	
Level 2	1469 (23.3)	538 (30.4)	
Level 3+	828 (13.1)	123 (6.9)	
**Hospital Profit**			
Non-Profit	4284 (67.6)	992 (55.8)	<0.001
For-Profit	1063 (16.8)	357 (20.1)	
Government	988 (15.6)	429 (24.1)	
**Percent Medicaid Discharges**			
<10%	304 (4.8)	83 (4.7)	<0.001
10-19.9%	1693 (26.7)	387 (21.8)	
20-49.9%	3850 (60.8)	1072 (60.3)	
≥50%	488 (7.7)	236 (13.3)	

CBSA=Core-Based Statistical Area

a. Generated from mixed model univariate logistic regression with admission as the outcome and the listed characteristic as the fixed effect with patient ID as a random intercept.
