# Supplementary material for: Patient and Hospital Characteristics Associated with Admission Among Patients With Minor Isolated Extremity Firearm Injuries: A Propensity-Matched Analysis
Source: Ann Surg Open. 2024 May 6;5(2):e430. doi: 10.1097/AS9.0000000000000430 (PMC11191909; doi:10.1097/AS9.0000000000000430)
Supplement: Supplementary file 6 [file as9-5-e430-s006.pdf]

Supplemental Table 5. Discharge disposition and Re-injury Status of Unmatched Admitted versus Non-admitted Patients with a Minor Isolated Extremity Firearm Injury Presenting to Hospitals in New York, Arkansas, Wisconsin, Massachusetts, Florida, and Maryland from 2016-2017 (N=8,151)

Outcomes	Not Admitted N=6,351, n(%)	Admitted N=1,800, n(%)	P-value[a]
**Discharge Disposition[b]**			
Home	5784 (91.1)	1372 (76.2)	
Transfer to Acute Care Facility	351 (5.5)	126 (7.0)	<0.001
Post-Acute Care Facility[c]/Home Health Care	99 (1.6)	244 (13.6)	
Other (Against Medical Advice, Died)[d]	117 (1.8)	58 (3.2)	
**Re-injury[e]**	877 (13.8)	233 (12.9)	0.35

a. P-values calculated using a mixed model logistic regression with admission status as the outcome and the listed characteristic as the lone fixed effect. Patient ID was fit as a random intercept.b. Categories consolidated to facilitate model convergence in cases of sparse cell sizes.

c. Post-Acute Care Facility includes discharge to hospice, rehabilitation, Long-term care hospital, psychiatric hospital, skilled nursing facility, or intermediate care facility.

d. Two patient encounters ended in death, both were admitted.

e. Re-injury was defined whether or not a patient presented to any hospital during the same calendar year for a new firearm injury.
