# Supplementary material for: Patient and Hospital Characteristics Associated with Admission Among Patients With Minor Isolated Extremity Firearm Injuries: A Propensity-Matched Analysis
Source: Ann Surg Open. 2024 May 6;5(2):e430. doi: 10.1097/AS9.0000000000000430 (PMC11191909; doi:10.1097/AS9.0000000000000430)
Supplement: Supplementary file 7 [file as9-5-e430-s007.pdf]

Supplemental Table 6: Multivariable Regression Modeling Inpatient Hospital Admission among Patients with a Minor Isolated Extremity Firearm Injury Presenting to Hospitals in New York, Arkansas, Wisconsin, Massachusetts, Florida, and Maryland from 2016-2017 (N=8,151)

	Odds Ratio[a]	95% Confidence Interval[b]
**Age (Years)**		
16-36	Ref	–
37-64	0.65	0.54, 0.78
>65	0.37	0.22, 0.61
**Sex**		
Male	Ref	–
Female	1.03	0.84, 1.28
**Race/Ethnicity**		
White (NH)	Ref	–
Black or African American (NH)	0.95	0.79, 1.15
Hispanic	1.29	0.99, 1.68
Other (NH)[c]	1.19	0.82, 1.72
**Insurance**		
Uninsured	Ref	–
Private	1.39	1.12, 1.70
Medicare	0.99	0.65, 1.49
Medicaid	0.99	0.82, 1.21
Other[d]	1.40	1.04, 1.90
**Injury Intent**		
Assault	Ref	–
Self-Inflicted	2.63	1.32, 5.26
Unintentional	0.52	0.45, 0.61
Undetermined	0.94	0.66, 1.35
Legal Intervention	0.22	0.09, 0.51
**Injury Type**		
Open Wound / Superficial Injury	Ref	–
Fracture / Dislocation	14.10	12.23, 16.26
Other	2.74	1.72, 4.34
**Elixhauser Comorbidity Score**	2.40	2.19, 2.63
**Trauma Center Level**		
Non-Trauma	Ref	–
Level 1	1.82	1.48, 2.24
Level 2	2.17	1.78, 2.66
Level 3+	1.08	0.81, 1.45
**Hospital Profit**		
Non-Profit	Ref	–
For-Profit	2.14	1.75, 2.63
Government	2.17	1.81, 2.59

a. Estimated from multivariable general estimating equation logistic regression including controlling for insurance, age, race, sex, injury intent, injury type, weighted Elixhauser

Comorbidity Index, trauma center designation, hospital profit type with observations clustered at the subject and hospital level

b. Confidence interval estimated by corresponding standard errors by use of generalized estimating equations

c. NH= Non-Hispanic; Other included Multiracial, self-described, Native, Asian/Pacific-Islander

d. Other Insurance included Worker's Compensation, CHAMPUS, CHAMPVA, Title V, and other government programs.
