# Supplementary material for: Patient and Hospital Characteristics Associated with Admission Among Patients With Minor Isolated Extremity Firearm Injuries: A Propensity-Matched Analysis
Source: Ann Surg Open. 2024 May 6;5(2):e430. doi: 10.1097/AS9.0000000000000430 (PMC11191909; doi:10.1097/AS9.0000000000000430)
Supplement: Supplementary file 8 [file as9-5-e430-s008.pdf]

Supplemental Table 7. Procedures of Unmatched Patients with a Minor Isolated Extremity Firearm Injury Presenting to Hospitals in New York, Arkansas, Wisconsin, Massachusetts, Florida, and Maryland from 2016-2017 (N=770)

Frequency	Procedure Description
65	Blood Transfusion
32	Repair Left Hand Tendon
27	Reposition Right tibia with internal fixation device (open)
19	Removal of foreign body or device from skin and subcutaneous tissue
18	Excision of right tibia open
18	Excision of left tibia (open)
15	Reposition left tibia with internal fixation device (open)
14	Repair right hand tendon
13	Excision of left lower leg subcutaneous tissue and fascia (open)
13	Extirpation of matter from left hand subq tissue and fascia (open)
12	Extirpation of Matter from Right Upper Leg Subcutaneous Tissue and Fascia, Open Approach.
12	Extirpation of Matter from Left Foot Subcutaneous Tissue and Fascia, Open Approach.
12	Extraction of Left Hand Subcutaneous Tissue and Fascia, Open Approach.
12	Excision of Left Finger Phalanx, Open Approach.
11	Extirpation of Matter from Left Lower Leg Subcutaneous Tissue and Fascia, Open Approach
10	Extirpation of Matter from Right Foot Subcutaneous Tissue and Fascia, Open Approach.
10	Reposition Left Lower Femur with Internal Fixation Device, Open Approach.
8	Extirpation of Matter from Right Lower Arm Subcutaneous Tissue and Fascia, Open Approach
8	Extraction of Left Upper Leg Subcutaneous Tissue and Fascia, Open Approach.
8	Excision of Left Ulna, Open Approach.
8	Excision of Left Metacarpal, Open Approach.
8	Excision of Right Metatarsal, Open Approach.
8	Reposition Right Upper Femur with Internal Fixation Device, Open Approach.
