# Supplementary material for: Patient and Hospital Characteristics Associated with Admission Among Patients With Minor Isolated Extremity Firearm Injuries: A Propensity-Matched Analysis
Source: Ann Surg Open. 2024 May 6;5(2):e430. doi: 10.1097/AS9.0000000000000430 (PMC11191909; doi:10.1097/AS9.0000000000000430)
Supplement: Supplementary file 9 [file as9-5-e430-s009.pdf]

**Supplemental Table 8. Patient Characteristics of Unmatched Admitted versus Non-admitted Patients with a Minor Isolated Extremity Firearm Injury Who Underwent a Subsequent Procedure Presenting to Hospitals in New York, Arkansas, Wisconsin, Massachusetts, Florida, and Maryland from 2016-2017 (N=770)**

| | **Admitted N=729** No (%) | **Not Admitted N=41** No (%) | **P-value[a]** |
|---|---|---|---|
| **Age (Years)** | | | |
| 16-36 | 487 (66.8) | 28 (68.3) | 0.85 |
| 37+ | 242 (33.2) | 13 (31.7) | |
| **Sex** | | | |
| Male | 661 (90.7) | 38 (92.7) | 0.68 |
| Female | 68 (9.3) | 3 (7.3) | |
| **Race/Ethnicity** | | | |
| White (NH) | 234 (32.6) | 20 (48.8) | |
| Black or African American/Other (NH) | 399 (55.5) | 13 (31.7) | 0.13 |
| Hispanic | 86 (12.0) | 8 (19.5) | |
| **Insurance** | | | |
| Medicaid/Medicare/Other[c] | 399 (55.0) | 17 (41.5) | |
| Private | 168 (23.1) | 10 (24.4) | 0.27 |
| Uninsured | 159 (21.9) | 14 (34.2) | |
| **Zip Code Income Quartile[d]** | | | |
| 1-2 | 544 (75.7) | 34 (85.0) | 0.24 |
| 3-4 | 175 (24.3) | 6 (15.0) | |
| **Intent** | | | |
| Assault | 307 (42.1) | 9 (22.0) | |
| Unintentional | 392 (53.8) | 29 (70.7) | 0.15 |
| Legal Intervention/Self-Inflicted/Undetermined | 30 (4.1) | 3 (7.3) | |
| **Injury Type** | | | |
| Fracture/Dislocation | 560 (79.2) | 22 (53.7) | 0.02 |
| Wound/Superficial Injury / Other | 147 (20.8) | 19 (46.3) | |
| **Extremity Abbreviated Injury Scale (AIS)** | | | |
| 1 | 547 (75.0) | 33 (80.5) | 0.47 |
| 2 | 182 (25.0) | 8 (19.5) | |
| **Elixhauser Comorbidity Score, Mean (SD)** | 1.0 (1.33) | 0.4 (0.66) | 0.04 |

NH=Non-Hispanic

a. Generated from mixed model univariate logistic regression with admission as the outcome and the listed characteristic as the lone fixed effect with subject ID as a random intercept.
b. Other included Multiracial, self-described, Native, Asian/Pacific-Islander
c. Other Insurance included Worker's Compensation, CHAMPUS, CHAMPVA, Title V, and other government programs.
d. Zip income quartile was a quartile classification of the estimated median household income of residents in the patient's ZIP Code.
