# Supplementary material for: Patient and Hospital Characteristics Associated with Admission Among Patients With Minor Isolated Extremity Firearm Injuries: A Propensity-Matched Analysis
Source: Ann Surg Open. 2024 May 6;5(2):e430. doi: 10.1097/AS9.0000000000000430 (PMC11191909; doi:10.1097/AS9.0000000000000430)
Supplement: Supplementary file 10 [file as9-5-e430-s010.pdf]

Sensitivity Table 9: Hospital Characteristics for Unmatched Admitted versus Non-admitted Patients with a Minor Isolated Extremity Firearm Injury Who Underwent a Subsequent Procedure Performed Presenting to Hospitals in New York, Arkansas, Wisconsin, Massachusetts, Florida, and Maryland from 2016-2017 (N=770)

	Admitted N=729 No. (%)	Admitted N=41 No. (%)	P-value[a]
**Hospital Bed Size**			
<100	21 (2.9)	6 (14.6)	
100-299	144 (20.0)	3 (7.3)	0.07
300-499	190 (26.4)	7 (17.1)	
≥500	365 (50.7)	25 (61.0)	
**Hospital Teaching Status**			
Teaching	554 (76.9)	32 (78.1)	0.88
Non-Teaching	166 (23.1)	9 (22.0)	
**Medical School Affiliated**			
Affiliated	494 (68.6)	25 (61.0)	0.37
Non-Affiliated	226 (31.4)	16 (39.0)	
**CBSA Type**			
Metro	693 (96.3)	38 (92.7)	0.32
Micro/Rural	27 (3.8)	3 (7.3)	
**Trauma Center Level**			
Non-Trauma	123 (17.1)	8 (19.5)	
Level 1	308 (42.9)	20 (48.8)	0.61
Level 2	216 (30.1)	8 (19.5)	
Level 3+	71 (9.9)	5 (12.2)	
**Hospital Profit**			
Non-Profit	448 (62.2)	28 (68.3)	
For-Profit	132 (18.3)	5 (12.2)	0.63
Government	140 (19.4)	8 (19.5)	
**Percent Medicaid Discharges**			
<19.9%	216 (30.0)	14 (34.2)	0.60
≥20.0%	504 (70.0)	27 (65.9)	

CBSA=Core-Based Statistical Area

a. Generated from mixed model univariate logistic regression with admission as the outcome and the listed characteristic as the lone fixed effect with subject ID as a random intercept.
