# Supplementary material for: Patient and Hospital Characteristics Associated with Admission Among Patients With Minor Isolated Extremity Firearm Injuries: A Propensity-Matched Analysis
Source: Ann Surg Open. 2024 May 6;5(2):e430. doi: 10.1097/AS9.0000000000000430 (PMC11191909; doi:10.1097/AS9.0000000000000430)
Supplement: Supplementary file 11 [file as9-5-e430-s011.pdf]

Supplemental Table 10: Multivariable Regression Modeling Inpatient Hospital Admission among Patients with a Minor Isolated Extremity Firearm Injury who underwent a Subsequent Procedure Performed Presenting to Hospitals in New York, Arkansas, Wisconsin, Massachusetts, Florida, and Maryland from 2016-2017 (N=770)

	Odds Ratio[a]	95% Confidence Interval[b]
**Race/Ethnicity**		
White (NH)	Ref	–
Black or African American/Other[c] (NH)	2.96	1.19, 7.37
Hispanic	1.22	0.45, 3.32
**Injury Type**		
Fracture/Dislocation	2.40	1.17, 4.92
Wound/Superficial Injury/Other	Ref	–
**Intent**		
Assault	Ref	–
Unintentional	0.43	0.17, 1.11
Legal Intervention/Self-Inflicted/Undetermined	0.27	0.05, 1.31
**Zip Code Quartile**		
1-2	Ref	–
3-4	2.18	0.85, 5.61
**Insurance**		
Medicaid/Medicare/Other[c]	1.40	0.63, 3.09
Private	1.70	0.66, 4.35
Uninsured	Ref	–
**Elixhauser Comorbidity Score**	1.92	1.28, 2.88
**Hospital Bed Size**		
<100	Ref	–
100-299	4.75	1.01, 22.31
300-499	2.71	0.78, 9.40
≥500	1.18	0.40, 3.50

a. Estimated from multivariable general estimating equation logistic regression controlling for race, injury type, intent, dichotomized zip code quartile, insurance, weighted Elixhauser Comorbidity Index, and hospital bed size with observations clustered by subject within hospital.
b. Confidence interval estimated by corresponding standard errors by use of generalized estimating equations
c. NH= Non-Hispanic; Other included Multiracial, self-described, Native, Asian/Pacific-Islander
